# Morphometry Difference of the Hippocampal Formation Between Blind and Sighted Individuals

**DOI:** 10.3389/fnins.2021.715749

**Published:** 2021-11-04

**Authors:** Ningning Pan, Ke Zheng, Yanna Zhao, Dan Zhang, Changxu Dong, Junhai Xu, Xianglin Li, Yuanjie Zheng

**Affiliations:** ^1^School of Information Science and Engineering, Shandong Normal University, Jinan, China; ^2^Master of Public Administration Education Center, Xinjiang Agricultural University, Xinjiang, China; ^3^College of Intelligence and Computing, Tianjin Key Lab of Cognitive Computing and Application, Tianjin University, Tianjin, China; ^4^Department of Mathematics and Computer Science, Eindhoven University of Technology, Eindhoven, Netherlands; ^5^Medical Imaging Research Institute, Binzhou Medical University, Yantai, China

**Keywords:** hippocampal formation, shape analysis, blindness, plasticity, spatial navigation

## Abstract

The detailed morphometry alterations of the human hippocampal formation (HF) for blind individuals are still understudied. 50 subjects were recruited from Yantai Affiliated Hospital of Binzhou Medical University, including 16 congenital blindness, 14 late blindness, and 20 sighted controls. Volume and shape analysis were conducted between the blind (congenital or late) and sighted groups to observe the (sub)regional alterations of the HF. No significant difference of the hippocampal volume was observed between the blind and sighted subjects. Rightward asymmetry of the hippocampal volume was found for both congenital and late blind individuals, while no significant hemispheric difference was observed for the sighted controls. Shape analysis showed that the superior and inferior parts of both the hippocampal head and tail expanded, while the medial and lateral parts constrained for the blind individuals as compared to the sighted controls. The morphometry alterations for the congenital blind and late blind individuals are nearly the same. Significant expansion of the superior part of the hippocampal tail for both congenital and late blind groups were observed for the left hippocampi after FDR correction. Current results suggest that the cross-model plastic may occur in both hemispheres of the HF to improve the navigation ability without the stimuli of visual cues, and the alteration is more prominent for the left hemisphere.

## Introduction

Spatial navigation is a complex process which depends on the confluence of various surrounding information including vision, auditory, and haptic cues (Lackner and DiZio, 2005; Fortin et al., 2008). Among the sensory inputs that generate the final sense of orientation, vision possesses the ability to reunite plenty of spatial information presented simultaneously. The “place cell” in the hippocampal formation (HF) can deal with the proximal and distal space information and update the motion cues continuously, which will fire with the occurrence of visual cues (Muller and Kubie, 1987; Saleem et al., 2018). As the central node of mnemonic circuitry, the HF is involved in the visual-spatial memory function and plays a critical role in the processing of space orientation (O’Keefe et al., 1998; Eichenbaum et al., 1999; Alme et al., 2014).

In contrast to the traditional studies that focus on the spatial navigation function of sighted individuals, structural alteration of human brain regions with the absence of visuals stimuli is particularly interesting (Gagnon et al., 2012). There is a pressing need for the blind individuals to develop alternative strategies that localize the surrounding objects using the remaining senses (Loomis et al., 2001; Giudice, 2018). It is interesting that congenitally blind subjects showed a better performance in the navigation tests which were conducted inside the life-size mazes or in the ecological environments than the blindfolded sighted individuals (Gagnon et al., 2010, 2012; Chebat et al., 2011). Stronger auditory sensitivity and navigation abilities were also found with early blind subjects as compared to the sighted controls (Dufour et al., 2005; Kupers and Ptito, 2014; Manescu et al., 2021). The HF is one of the regions that produce plastic changes (cross-modal plastic) in humans with the help of extensive visual-spatial navigation training (Lazzouni and Lepore, 2014; Chebat et al., 2018). Alterations in the right HF has been observed in early blind individuals using volume analysis method (Chebat et al., 2007). Both the frontal lobe and HF were activated during the learning process with the stimuli of auditory based on the studies using functional Magnetic Resonance Imaging (fMRI) data (Crottaz-Herbette et al., 2005; Naumer et al., 2009). Delineating the detailed morphometry changes of the HF is essential for the understanding of the plastic phenomenon during the navigation process without the stimuli of visual cues.

However, the HF is a complex brain region comprised of several substructures (Krogsrud et al., 2014; Romero et al., 2017). The volume of the whole HF could show no significant variation as increase or decrease of specific subfields may occur together (Fortin et al., 2008). Volumetric analysis used in previous studies thus might not be sensitive enough to detect detailed alterations of the HF between blind individuals and sighted controls. Shape analysis, on the other hand, has been performed to observe the local changes of brain regions in diverse communities (Lai et al., 2009; Shi et al., 2011; Tae et al., 2011; Ng et al., 2014; Gahm and Shi, 2016). Similar research was proposed in previous publications on the development of fetal brain regions including the HF and cerebellum (Ge et al., 2015; Xu et al., 2020). As to the blind hippocampi, relevant results demonstrated that the anterior part of the right HF is larger with blind individuals compared to the sighted controls based on shape analysis method (Leporé et al., 2009). On the contrary, significantly smaller regions was found in the posterior part of the right hippocampi, which indicates the heterogeneity of different subregions of the HF (Leporé et al., 2009). However, different patterns of dysfunction or different blinding timing (congenital and late blindness) were considered together in previous studies using shape analysis method, which may develop significant deviation of the HF from its normal surface among different onset patterns of blindness. The detailed morphometry alterations of the HF for either congenital or late blind individuals are still not well described.

In this study, 16, 14, and 20 of congenital blind, late blind, and sighted individuals were recruited, respectively, to calculate the morphometry difference of the HF between the blind and sighted individuals. Shape analysis based on Riemannian metric optimization on surfaces (RMOS) method was used to characterize the regional alterations of the HF that caused by blindness (Gahm and Shi, 2016). We hypothesized that current research may provide detailed representation of the morphometry alterations of the blind HF compared to that of the sighted controls. Different onset timing of blindness may exhibit diverse plastic patterns. Plastic changes may occur in both hemispheres of the blind HF, which may provide additional contributions for the understanding of cross-model plastic of human brain without the stimuli of visual cues.

## Materials and Methods

### Participants

Thirty blind subjects (16 congenital blind and 14 late blind) and twenty sighted controls were recruited from Yantai Affiliated Hospital of Binzhou Medical University. The current cohort had been partly used to delineate the alteration of functional brain network in our previous publication (Li et al., 2019). The basic information of the current cohort is shown in Table 1. All blind subjects were diagnosed cataracts or retinal pigment degeneration based on the consensus of two professional ophthalmologists. No neurological disabilities except for the visual deprivation of the blindness were considered as one of the inclusion criteria in the current study. The approval from the Institutional Review Board of Binzhou Medical University was acquired for the conduction of current study. Written informed consent was obtained from each subject after understanding the purpose of this study.

**TABLE 1:** The demographic information of the cohort.

Group	Con	Cong	*P* value (Cong vs Con)	Late	*P* value (Cong vs Con)
Number (N)	20	16	\	14	\
Age (year)	21.97 ± 1.16	22.40 ± 2.12	0.43	22.18 ± 1.67	0.66
Gender (M/F)	10/10	12/4	0.13	11/3	0.09
Onset time (year)	\	0	\	10.21 ± 5.52	\

*Two tailed student t-tests and Chi-square tests were conducted for comparison of age and gender distribution between blind and sighted groups, respectively. Con, control group; Cong, congenital blind group; Late, late blind group; M, male; F, female.*

### Data Acquisition

T1 weighted MR images were acquired with a 3.0 T Siemens Skyra scanner at Yantai Affiliated Hospital of Binzhou Medical University. 3D MPRAGE sequence was used and the parameters are as follows: repetition time: 1,900 ms, echo time: 2.52 ms, inversion time: 1,100 ms, voxel size: 1 × 1 × 1 mm^3^, matrix size: 256 × 256, flip angle: 90°.

### Segmentation of the Hippocampal Formation

**FIGURE 1:**
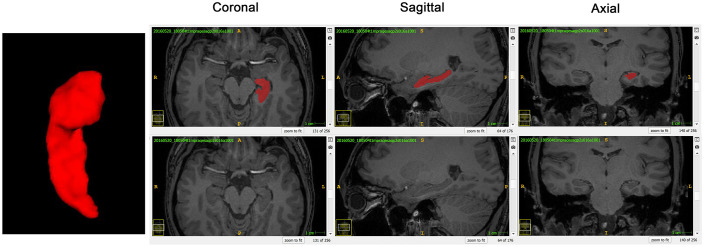
A normal sighted subject was taken as the example to show segmentation and surface reconstruction of the left hippocampal formation (HF) using the ITK-SNAP software. The 3D reconstructed HF and the coronal, sagittal, and axial planes were displayed from left to right, respectively.

The HF was manually segmented by one anatomist with the help of ITK-SNAP software (Yushkevich et al., 2006), referenced by the previous studies and histological atlas (Ge et al., 2015; Yushkevich et al., 2015; Adler et al., 2018). Compared with fetal HF, segmentation of the adult HF is easier and faster as the hippocampal tail is far away from corpus callosum and cingulate gyrus. The only challenge is the boundary between amygdala and hippocampal head as both of them are closely located in the temporal lobe (Konrad et al., 2009). The CSF between hippocampal head and amygdala was used for the boundary determination in the current segmentation protocol (Figure 1).

Twenty subjects were selected randomly from the whole cohort 3 months later and re-segmented manually by another anatomist to check the reliability of the above segmentation. Dice’s coefficient was calculated based on the intraclass consistency of the two segmentations, which was also used in our previous publication (Ge et al., 2015).

### Volume Analysis

Absolute volume of the HF was calculated based on the binary masks acquired from the segmentation section. The relative volume of the HF was calculated *via* the following formula: V_relative_ = V_absolute_/ICV, where the intracranial volume (ICV) for each subjects was acquired based on the Bet process results in FSL software (Jenkinson et al., 2012).

### Shape Analysis

Prior to perform shape analysis, the hippocampal templates of both hemispheres were established separately using *buildtemplateparelle.sh* on ANTs software (Avants et al., 2009). Default options of ANTs software were selected for this process.

Surface meshes of the HF for all subjects as well as the template were then acquired from the binary masks. All surface meshes were re-meshed to 1,000 vertices based on the size of the HF as well as considering the computational cost. The individual re-meshed surface was mapped to the re-meshed template based on RMOS algorithm (Gahm and Shi, 2016). Similar to the previous studies (Ge et al., 2015; Xu et al., 2020), the thickness of left and right hippocampi was measured separately and each vertex on the mapped surface was calculated by the distance from the surface vertex to the medial core of the HF. More detailed description of the shape analyses method can be found in our previous publication (Ge et al., 2015).

### Statistical Analysis

Two tailed student *t*-test was conducted for the comparison of the hippocampal volume between the blind and sighted individuals, as well as the hemispheric difference within each group. The same *t*-test method was conducted on the thickness of the shape surface points to calculate the regional difference between the blind and sighted groups using Surfstat software on the MATLAB platform (Worsley et al., 2009). The volume and shape differences between the congenital/late blind individuals and sighted controls were calculated separately. The false discovery rate (FDR) correction was applied for the correction of multiple comparisons for all statistical tests. Significance for all statistical analysis was set to *p* < 0.05.

## Results

### Study Cohort Characteristics and Reliability of the Segmentation

Demographic and clinical characteristics of the study cohort are presented in Table 1. No significant difference of related characteristics (age and gender distribution) is found between the blind individuals (either congenital and late blindness) and sighted controls.

The average Dice’s coefficients are 0.9233 for the left hippocampi and 0.9352 for the right hemisphere, which demonstrate high reproducibility of the segmentation protocol in the current study.

### Volume Analysis

**FIGURE 2:**
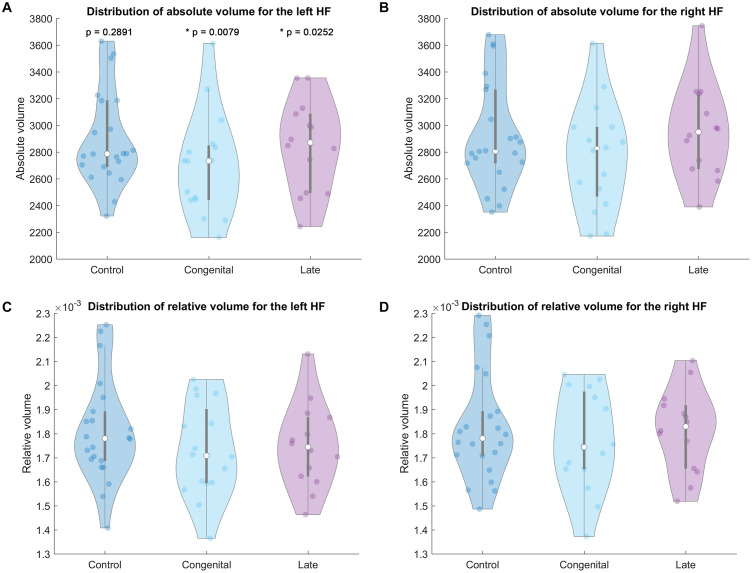
Violin plot of the hippocampal volumes for each group. *Top row* absolute volume of the left **(A)** and right **(B)** HF. *Bottom row* relative volume of the left **(C)** and right **(D)** HF. *P* values in the top left A part mean the hemispheric difference of the absolute volumes for each group, p < 0.05 labeled with * as statistically significant.

Absolute and relative hippocampal volume information of the current cohort is shown in Table 2 and Figure 2. No significant difference of the hippocampal volumes (either absolute or relative volume) between blind (either congenital or late blind) and sighted subjects was found for both hemispheres (*p* > 0.05, Table 2). Both congenital blind (*p* = 0.0079) and late blind (*p* = 0.0252) individuals show significantly rightward asymmetry of the absolute hippocampal volume, whereas control group does not show asymmetry difference (*p* = 0.2891).

**TABLE 2:** Volume information of the current cohort.

	Hemisphere	Con	Cong	*P* value (Cong vs Con)	Late	*P* value (Late vs Con)
Absolute	L (*10^3^mm^3^)	2.89 ± 0.35	2.70 ± 0.38	0.1195	2.85 ± 0.34	0.7262
	R (*10^3^mm^3^)	2.92 ± 0.39	2.77 ± 0.40	0.2318	2.96 ± 0.35	0.8030
Relative	L (*10^–3^)	1.80 ± 0.21	1.70 ± 0.19	0.2406	1.70 ± 0.17	0.3847
	R (*10^–3^)	1.80 ± 0.22	1.80 ± 0.20	0.4259	1.80 ± 0.17	0.8568

*L, left; R, right; Con, control group; Cong, congenital blind group; Late, late blind group. *p* < 0.05 as statistically significant.*

### Shape Analysis

#### Congenital Blind Versus Control Group

**FIGURE 3:**
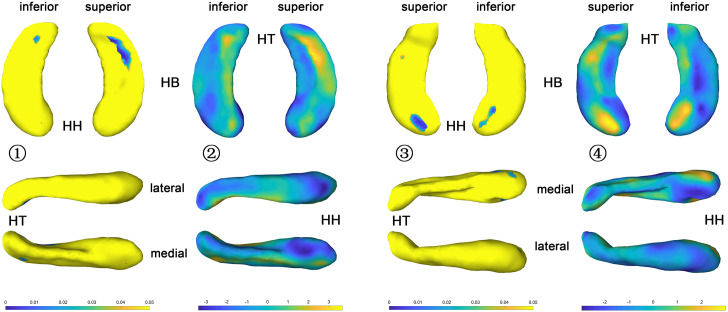
*P* value and *t* value maps of congenital blind subjects compared with control group (uncorrected), from left to right columns are (1) *p* value map of left HF, (2) *t* value map of left HF, (3) *p* value map of right HF, and (4) *t* value map of right HF, respectively, *p* < 0.05 was considered as statistically significant. HH, hippocampal head; HB, hippocampal body; HT, hippocampal tail.

**FIGURE 4:**
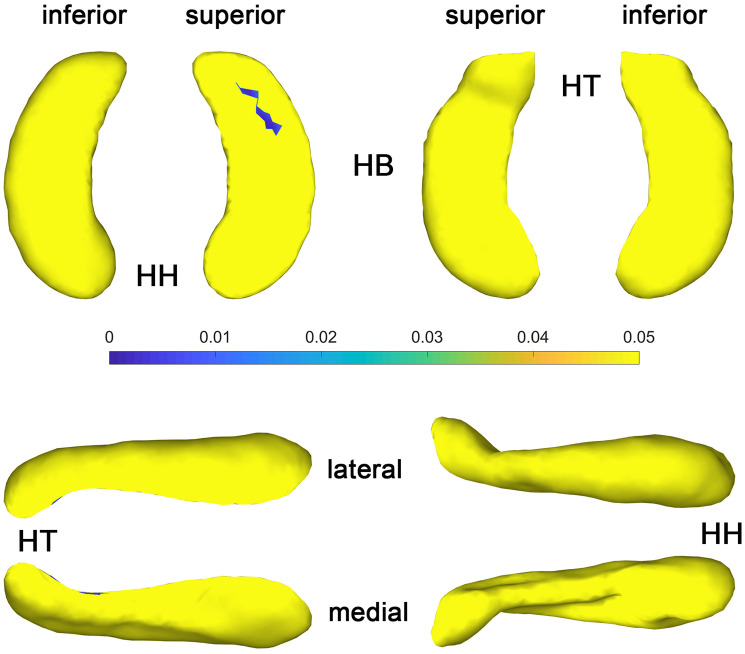
*P* value maps of congenital blind subjects compared with control group after FDR correction, from left to right columns are *p* value maps of left and right HF, respectively, *p* < 0.05 was considered as statistically significant. HH, hippocampal head; HB, hippocampal body; HT, hippocampal tail.

The statistical results (*p*-value and *t*-value maps) that represent the comparison between congenital blind and control groups are shown in Figures 3, 4. Compared to the sighted control group, congenital blind individuals exhibit expansion of the shape surface mainly in the superior and inferior parts, while show contraction in the medial and lateral parts of both the hippocampal head and tail for the left hippocampi (Figure 3-2). The increase or decrease of the thickness is not prominent in the region of hippocampal body. However, only the superior and inferior parts of the hippocampal tail expand significantly (*p* < 0.05, Figure 3-1). As to the right hippocampi, similar results are found from the *t*-value maps with the high thickness located in the superior and inferior parts of the hippocampal head and tail for the congenital blind group (Figure 3-4). The alteration of the hippocampal body is still not prominent for the right HF. Unlike the left hippocampi, the increased thickness of superior and inferior parts of the hippocampal head are statistically significant for the right hippocampi (*p* < 0.05, Figure 3-3). After FDR correction, the reserved regions that show significant alteration of the thickness between the congenital blind and sighted control groups only focus on the superior part of the hippocampal tail for the left hippocampi (Figure 4).

#### Late Blind Versus Control Group

**FIGURE 5:**
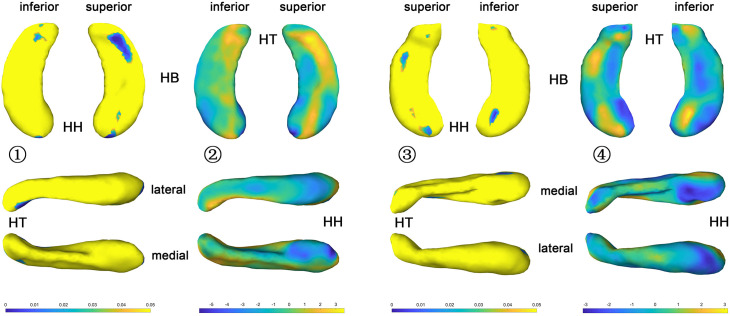
*P* value and *t* value maps of late blind subjects compared with control group (uncorrected), from left to right columns are (1) *p* value map of left HF, (2) *t* value map of left HF, (3) *p* value map of right HF, and (4) *t* value map of right HF, respectively, *p* < 0.05 was considered as statistically significant. HH, hippocampal head; HB, hippocampal body; HT, hippocampal tail.

**FIGURE 6:**
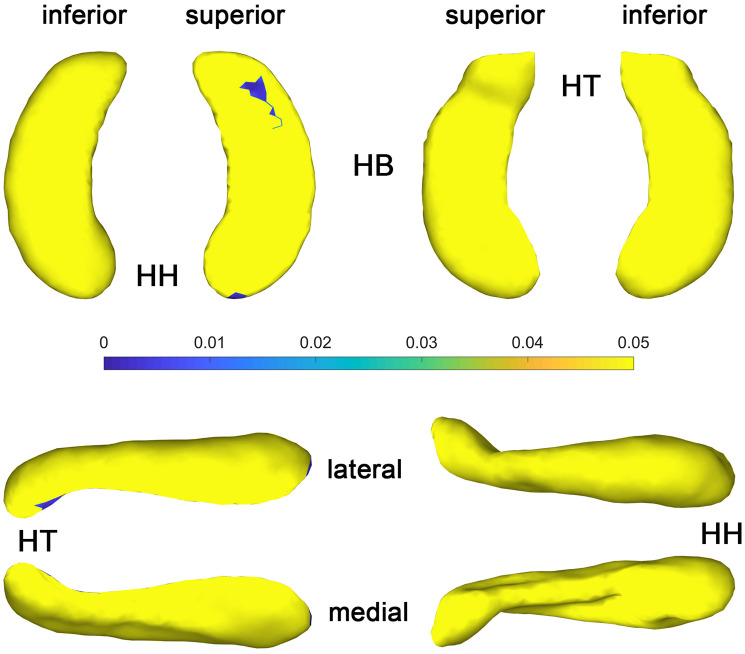
*P* value maps of late blind subjects compared with control group after FDR correction, from left to right columns are *p* value maps of left and right HF, respectively, *p* < 0.05 was considered as statistically significant. HH, hippocampal head; HB, hippocampal body; HT, hippocampal tail.

The *t*-value maps that represent the difference of the hippocampal thickness between late blind and control groups and the *p*-value maps that show the significance of the difference are shown in Figure 5. Compared to the control group, late blind individuals show expansion in the superior and inferior parts of nearly the whole HF, while contraction appears in the medial and lateral parts of the hippocampal head for the left hippocampi (Figure 5-2). *P*-value maps show nearly the same result between the late blind and sighted control groups as compared to the comparison between the congenital blind and sighted control groups. Only the superior and inferior parts of the hippocampal tail of the left hippocampi are statistically significant (Figure 5-1). Small area of the anterior area of the hippocampal head also shows significant increase of the hippocampal thickness. For the right hippocampi, similar results are found from the *t*-value maps with the higher thickness located in the superior and inferior areas of both the hippocampal head and tail for the late blind group compared to the control group. The alteration of the hippocampal body is still not prominent for the right HF (Figure 5-4). Unlike the left hippocampi, the increased thickness of superior and inferior areas of the hippocampal head, and the superior part of the hippocampal tail are statistically significant for the right hippocampi (Figure 5-3). After FDR correction, the significant regions only focus on the superior part of the hippocampal tail and the most anterior part of the hippocampal head for the left hippocampi, whereas no significant different are found with the right hemisphere (Figure 6).

## Discussion

Volume analysis has been conducted in numerous studies to observe the morphometry changes of the HF (Woolard and Heckers, 2012; Yushkevich et al., 2015; Chen et al., 2018). Reduced volume was found with the right HF, while no significant difference was discovered with the left hemisphere of the congenital blind subjects compared to the sighted controls (Chebat et al., 2007). However, congenitally blind subjects performed better in the navigation tests that conducted inside the life-size mazes or in the ecological environments than the blindfolded sighted individuals (Gagnon et al., 2010, 2012; Chebat et al., 2011). When limiting the access to allothetic cues such as temperature and echolocation, blind subjects will lose their competitive advantages compared with controls in spatial navigation tasks (Gagnon et al., 2010). In addition, volume of the posterior HF is positively associated with the time of duration as the taxi drivers, while anterior HF shows negative correlation (Maguire et al., 2000). The posterior HF may expand significantly in response to store plenty of surrounding spatial information (Maguire et al., 2003). Moreover, different hemispheres of the cuneus was found significantly activated in distinct sound-mediated pattern-spatial recognition tasks for blind subjects compared to the normal controls (Arno et al., 2001; Voss et al., 2008). The inferior parietal cortex can integrate the tactile inputs to match visualization, auditory, and spatial information (Weeks et al., 2000; Collignon et al., 2007). Above studies indicated that the cross-modal plasticity or visual compensation may occur on the subregions of the HF as well as other brain regions to build effective spatial representations of the environment after the loss of visual stimuli.

However, recent research demonstrated no significant association between the volume of the HF and navigation function in the typical population (Weisberg et al., 2019). Similar results were also found in the current study with no significant difference between the blind (either congenital or late blindness) and sighted individuals of the hippocampal volume (either absolute or relative volume) (Table 2). Only rightward asymmetry of the hippocampal volume is found for the blind subjects, which indicates that the arisen of blindness may prompt the occurrence of hemispheric difference of the HF. Together with previous studies, there are debates of using the volumetric analysis to detect the morphometry changes of the blind HF. One possible reason is the volume analysis can only reflect the total representation of the brain (sub)regions, which is insufficient to delineate the subtle changes of the subfields of the relatively small regions such as the dentate gyrus, subiculum, and CA areas of the HF. However, the subfields of the HF are heterogeneous in function (Libby et al., 2012; Aanes et al., 2019). The increase of one subfield and the decrease of another one may occur simultaneously and lead to nearly no volumetric changes of the whole (sub)region. Another possibility is that the significantly correlation between the hippocampal volume and navigational ability is only detectable in extreme groups (Weisberg et al., 2019). It is hard to detect the differences of the ordinary blindness compared to the normal controls based on the volume analysis method. The delineation of the volume, on the other hand, may be influenced by the segmentation protocol, the resolution and field intensity of the MRI scanning, as well as the complexity of specific region that related to the research topics (Despotović et al., 2015). The hippocampal volumes thus may be insufficient to be regarded as a biological marker for the supervision of navigation ability (Weisberg et al., 2019; Clark et al., 2020).

The shape analysis method that widely used recently is a more detailed morphometry analysis tool, which can be regarded as the powerful supplement to the traditional volume analysis method. Subtle changes of the small brain regions such as development of the fetal HF has been observed with the help of shape analysis method in the previous study (Ge et al., 2015). As the hippocampal surface is heterogeneous in function, distinct structural changes may occur in diverse hippocampal subfields with the arisen of blindness. Patterns of the hippocampal shapes of blind and sighted individuals have been described based on the conformal metric optimization on surfaces (CMOS) registration algorithm (Leporé et al., 2009). Compared to the control group, significantly increased thickness of the anterior part and decreased thickness of the posterior part of the right HP were found with blind individuals. On the contrary, no significant difference was observed for left HP between the blind and sighted controls (Leporé et al., 2009). However, the congenital and late blind subjects were considered together for the statistical analyses in the former study, which may introduce bias of the final conclusion. In the current research, we recruit congenital blind, late blind, and sighted normal control individuals to observe the volume and surface changes that caused by blindness. Compared to the sighted individuals, increased thickness of the superior and inferior parts, and decreased thickness of the medial and lateral areas of the hippocampal head and tail for both hemispheres were found for the blind subjects (Figures 3, 5). The increase of the thickness is significant in the hippocampal tail for the left hippocampi, and in the hippocampal head for the right hippocampi (uncorrected). As we all known, the right HF is related to topographic orientation and spatial memory, whereas the left hippocampus is associated with episodic and autographical event memory (Berthoz, 1997; Maguire, 2001; Viard et al., 2007). Enlarged anterior HF has been related to the enhanced navigation ability with congenital blind subjects (Fortin et al., 2008). Similarly, the right para-hippocampus and visual cortex of the trained blind individuals exhibited more activation than the blindfolded sighted controls during the route recognition task (Kupers et al., 2010). The expansion of superior and inferior regions of the hippocampal head in the current research indicate that the blind individuals may produce more “place cells” to improve the navigation ability (Save et al., 1998; Wolbers and Hegarty, 2010; Lisman et al., 2017). However, only the left hippocampi show significant increase of the thickness after FDR correction in the current research (Figures 4, 6). The left HF mediates learning by binding different sensory modalities, such as the sound and distance information (Gabrieli et al., 1997; Gonzalo et al., 2000; Stark and Squire, 2001; Small, 2002; Norman and O’Reilly, 2003; Preston and Eichenbaum, 2013). The left HF has also been involved in the integration of auditory-visual stimuli (Calvert et al., 2001). The significant increase of the left HF in the current study indicates that the blindness may prompt the plastic of left hippocampi to acquire the non-spatial signal to improve the navigation ability (Chan et al., 2012).

Previous study found that the congenital blind individuals performed better than the late blind and the sighted blindfolded groups (Passini et al., 1990). Compared to the late blind individuals, enhanced odorant localization abilities was also observed with congenitally blind subjects (Manescu et al., 2021). However, the alterations of the congenital and late blind individuals are nearly the same, and the right hippocampi exhibit more areas with the increased thickness in the current research (Figures 3, 5, *t*-value maps). One possibility is the hippocampal plastic is a long-time course and follows the “Law of Use and Disuse” features of the human organs. The regions associated with straightforward navigation ability (right hippocampi) of the congenital blind subjects may be altered with the stimuli of diverse sensory except visual cues, and the alteration may be inadequate as compared to the adventitiously totally blind individuals. There may be a sharp alteration of the HF with the late blind individuals to deal with the deficiency of visual cues. Another possibility is the relatively small sample size used in the current research, which may be insufficient to detect the detailed difference between the congenital and late blind subjects.

This study has several limitations. The uneven gender distribution with more male subjects in either the congenital blind group or the late blind group may introduce some unexpected influence on the statistical results. This is why we did not consider the gender difference in the current research. In addition, the onset time point of the blindness is with a large span for the late blind subjects, which may also influence the statistical results. It is well known that the alteration of the brain regions may last an extremely long time to be detectable. Late blind subjects with diverse time of duration of the blindness may exhibit different alteration patterns of the hippocampal morphometry. Recruiting the subjects with nearly the same onset time point of the blindness is therefore essential to delineate the features of the hippocampal plastic. On the other hand, there are few studies to conduct longitudinal analysis on the blind HF. To further discover the underlying mechanism of the morphometry alterations of the blind HF, more samples should be recruited and longitudinal analysis based on the follow up scans should also be considered in the future studies.

## Conclusion

Current study elucidated the morphometry difference between the blind individuals and sighted controls based on the volume and shape analysis methods. Significant increases of the hippocampal tails for the left hippocampi and hippocampal head for the right hippocampi were observed with the blind individuals compared to the control group. Current results indicate that the plastic of the HF is heterogenous and may occur in distinct subregions of the left and right HF to maintain the navigation ability without the stimuli of visual cues.

## Data Availability Statement

The raw data supporting the conclusion of this article will be made available by the authors, without undue reservation.

## Ethics Statement

The studies involving human participants were reviewed and approved by Institutional Review Board of Binzhou Medical University. The patients/participants provided their written informed consent to participate in this study.

## Author Contributions

NP: drafting the manuscript, analysis and interpretation of data. KZ: data analysis. YaZ: revising the manuscript critically for important intellectual content. DZ: review of manuscript. CD: analysis and interpretation of data. JX: statistical analysis. XL: acquisition of data. YuZ: study conception and obtain funding, design of study, and review of manuscript. All authors contributed to the article and approved the submitted version.

## Conflict of Interest

The authors declare that the research was conducted in the absence of any commercial or financial relationships that could be construed as a potential conflict of interest.

## Publisher’s Note

All claims expressed in this article are solely those of the authors and do not necessarily represent those of their affiliated organizations, or those of the publisher, the editors and the reviewers. Any product that may be evaluated in this article, or claim that may be made by its manufacturer, is not guaranteed or endorsed by the publisher.
